# Concentrating of Sugar Syrup in Bioethanol Production Using Sweeping Gas Membrane Distillation

**DOI:** 10.3390/membranes9050059

**Published:** 2019-05-01

**Authors:** Mohammad Mahdi A. Shirazi, Ali Kargari

**Affiliations:** 1 Membrane Industry Development Institute, Tehran 1587856614, Iran; mmahdiashirazi@gmail.com; 2 Membrane Processes Research Laboratory (MPRL), Department of Chemical Engineering, Amirkabir University of Technology (Tehran Polytechnic); Tehran 1591634311, Iran

**Keywords:** bioethanol, sweeping gas membrane distillation, SGMD, glucose, permeate flux, optimization

## Abstract

Membrane distillation (MD) is a relatively new and underdeveloped separation process which can be classified as a green technology. However, in order to investigate its dark points, sensitivity analysis and optimization studies are critical. In this work, a number of MD experiments were performed for concentrating glucose syrup using a sweeping gas membrane distillation (SGMD) process as a critical step in bioethanol production. The experimental design method was the Taguchi orthogonal array (an *L*_9_ orthogonal one) methodology. The experimental results showed the effects of various operating variables, including temperature (45, 55, and 65 °C), flow rate (200, 400, and 600 ml/min) and glucose concentration (10, 30, and 50 g/l) of the feed stream, as well as sweeping gas flow rate (4, 10, and 16 standard cubic feet per hour (SCFH)) on the permeate flux. The main effects of the operating variables were reported. An ANOVA analysis showed that the most and the least influenced variables were feed temperature and feed flow rate, each one with 62.1% and 6.1% contributions, respectively. The glucose rejection was measured at 99% for all experiments. Results indicated that the SGMD process could be considered as a versatile and clean process in the sugar concentration step of the bioethanol production.

## 1. Introduction

The increasing global energy demand and its ensuing crisis, as well as the highlighting of major environmental challenges in recent years have led to considerable interest for substituting hydrocarbon fuels with renewable and sustainable energy sources [1,2,3,4]. Several alternatives have been explored, including a number of carbon-free sources in an attempt to replace hydrocarbon fuels [5].

Among the different renewable energies, biofuels, and in particular bioethanol as a clean fuel, have gained a great deal of attention [5,6,7]. Bioethanol can be produced from a wide range of renewable materials such as cellulose, algae, sorghum, and corn biomass. Burning bioethanol, either in place of gasoline or in the form of an ethanol–gasoline, can reduce global warming emissions up to 80%. This can entirely eliminate the release of acid rain [8]. Moreover, bioethanol can be mixed with gasoline for transportation purposes. This technique has been widely used in several countries such as Brazil. However, bioethanol processing and production involves a number of steps (e.g., pretreatment, fermentation, recovery, and refining), and it should be noted that bioethanol can be more significantly beneficial for the environment with improvements to this process, by reducing the required amount of energy. Membrane processes have gained a great deal of attention for their various applications [9,10] including its role in bioethanol processing [11]. This is attributed to their lower energy requirements, lower labor costs, less use of land, and remarkable operational flexibility.

Every bioethanol generation procedure involves saccharification and fermentation processes [5]. However, the concentration of the fermentable sugar in the prehydrolyzates is an important issue. In other words, low sugar concentration can lead to lower bioethanol production, which translates to higher costs and energy consumption in subsequent steps in the process [8]. As a result, the prehydrolyzate should be concentrated (to increase the sugar content) for enhancing the effectiveness of the fermentation step for bioethanol production. Various membrane processes have been used for concentrating sugar syrups such as nanofiltration (NF), reverse osmosis (RO) [12], and membrane distillation [13].

The membrane distillation (MD) process is a relatively new and underdeveloped separation method [10]. This non-isothermal membrane process involves the transport of vapor molecules through the pores of a hydrophobic microporous membrane. Membrane distillation is driven by the vapor pressure difference provided by a temperature difference between the sides of the applied membrane [14]. The hydrophobic characteristic of the applied membranes allows only vapor molecules to pass thorough the pores while holding back the liquid phase. One of the highlighted advantages of the MD process is the relatively considerable permeate fluxes obtained at moderate feed temperatures [15]. In MD, the hydrophobic microporous structure of the membrane plays no role in selectivity for the target component (from a macroscopic point of view) and acts as an interface for the vapor–liquid equilibria (from a microscopic point of view).

In this process, different configurations have been used to impose the driving force and provide the permeate flux. These include direct contact MD (DCMD), air-gap MD (AGMD), vacuum MD (VMD), and sweeping gas membrane distillation (SGMD). Table 1 illustrates a comparison and description of each MD configuration. It is worth noting that, the SGMD seems to be more suitable in processes where permeate is not the target product and can be vented such as concentrating of aqueous solutions containing a non-volatile solute. The application of various MD configurations for different purposes including desalination and bioethanol processing [10,11], the application of atomic force microscopy (AFM) for characterizing the MD membranes [16], and a comprehensive study on polytetrafluoroethylene (PTFE) membranes for MD desalination [17] have been studied comprehensively.

In this work, the sensitivity analysis of the SGMD process for a new application in bioethanol processing, i.e., concentrating the glucose syrup, was investigated. To optimize the experiments, it was obviously necessary to identify which variables were more influential on the target parameter (i.e., the permeate flux in this work). Thus, the Taguchi experimental design (Qualitec-4) was used. Taguchi’s approach first defines a set of orthogonal arrays and second devises a standard method for analysis of results. Two important issues should well be pleased by the Taguchi method including the number of trials and the conditions for each one. One of the most important advantages of this method is the significant reduction in time and number of experiments required for obtaining the optimum operating conditions. Moreover, it determines which variable has more influence, and which has less. Therefore, the optimum level for each factor can be determined. Afterward, the confirmation of the predicted value (permeate flux in this work) should be performed.

## 2. Materials and Methods

### 2.1. Materials

A commercial flat sheet hydrophobic membrane made of polytetrafluoroethylene (PTFE) (Millipore, USA) with ~170 cm^2^ effective area was used. Table 2 presents the specifications of the applied membrane. Pure water (double distillated water) and analytical reagent grade glucose (purity >99.4%, BASF, Germany) were used for preparation of feed samples with the desired concentrations (10, 30, and 50 g/l). Dried air (after filtration) was selected as the sweeping gas.

### 2.2. SGMD Experimental Apparatus

**Figure 1:**
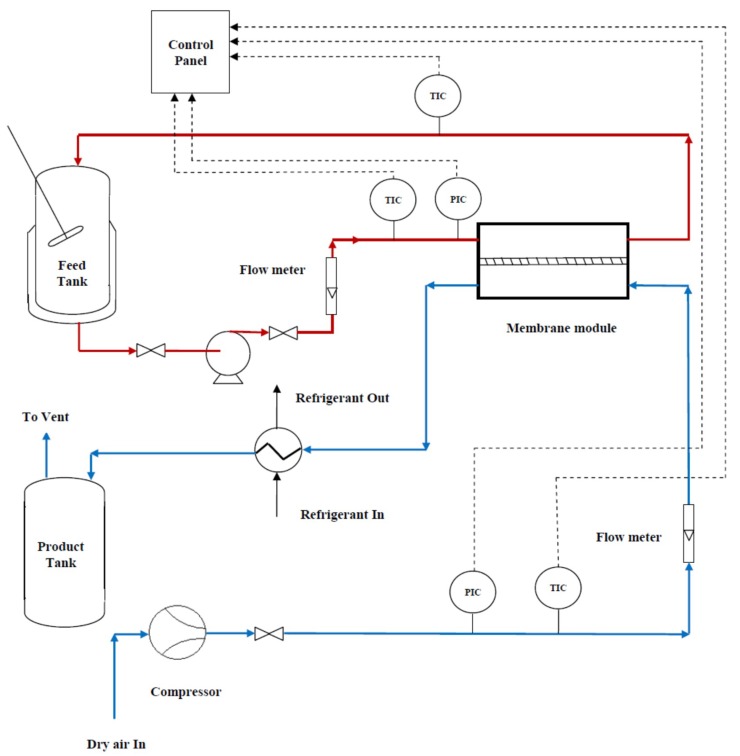
The general scheme of the experimental sweeping gas membrane distillation (SGMD) setup. The system includes the hot side (with red lines): a jacket tank equipped with an over-head mixer and a Pt-100 thermal sensor, a diaphragm pump for recirculation of the feed, flowmeter, the SGMD module equipped with a polytetrafluoroethylene (PTFE) (0.22 µm) membrane and four Pt-100 thermal sensors for entrance and exit points; and the cold side (with blue lines): an oil-free compressor for proving the SG, a flow-meter for adjusting the SG flow, feed tank, and refrigerator system for condensing the permeate vapor.

The applied membrane was placed in a cross-flow plate and frame module with 130 × 130 mm^2^ dimensions. The feed and permeate channels depth were 2 mm. A heating bath equipped with a PID controller (Autonics, Korea) and Pt-100 temperature sensors were used for the feed temperature control. Four sensors were located as close as possible to the inlet and outlet sections of the module and another one was located inside the feed tank. A diaphragm pump (So~Pure, Korea) was used to re-circulate the hot feed in a closed loop of “the feed tank-MD module-feed tank”. A compressor (an oil-free GAST compressor, USA, to ensure an oil-free air stream) supplied the sweeping gas (SG) flow. A cooling system was used to condense the vapor molecules. Figure 1 shows the general scheme of the used MD system.

### 2.3. Experimental Procedure and Analysis

In each experiment, samples were taken from the feed, permeate, and concentrate every 30 minutes, and analyzed for glucose content by using the glucose oxidase colorimetric method, which has been described in a previous work [18]. The performance of the SGMD process was evaluated based on two major parameters: the permeate flux and the selectivity. Flux was defined as the mass or volume (*L*) collected in the permeate channel per the membrane’s effective area (*m*^2^) and the time (*h*) of the experiment. Permeate flux and selectivity were calculated using the following equations:

(1) $$F_{D}=\frac{V}{A\cdot t}$$

(2) $$S=\frac{y_{e}(1-x_{e})}{x_{e}(1-y_{e})}$$

where *V* is the condensed water in the permeate channel, *A* is membrane’s effective area, and *t* is the time interval, respectively. The *y_e_* and *x_e_* symbols refer to the mass fractions of glucose in the permeate and the feed streams, respectively.

**Figure 2:**
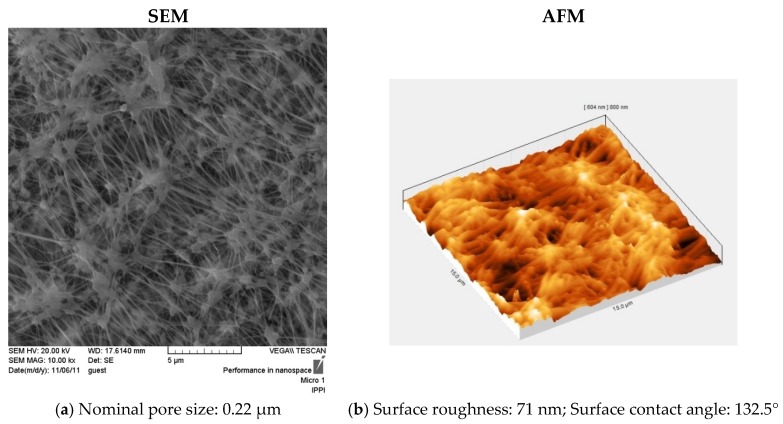
The scanning electron microscopy (SEM) (with 5-µm scale-bar) and atomic force microscopy (AFM) (with 15 µm × 15 µm scanning size) images of the PTFE membrane (with 0.22-µm pore size) used in this work.

Scanning electron microscopy (SEM) (VEGA, TESCAN, Czech Republic), atomic force microscopy (AFM) (DUALSCOP 95-200E, DEM, Denmark), and contact angle test (KRUSS G-10, Germany) were used for morphology observations of the applied membrane, based on the procedures described in the previous work [16]. Figure 2 shows the SEM and AFM images of the used membrane in this work.

## 3. Results

**Figure 3:**
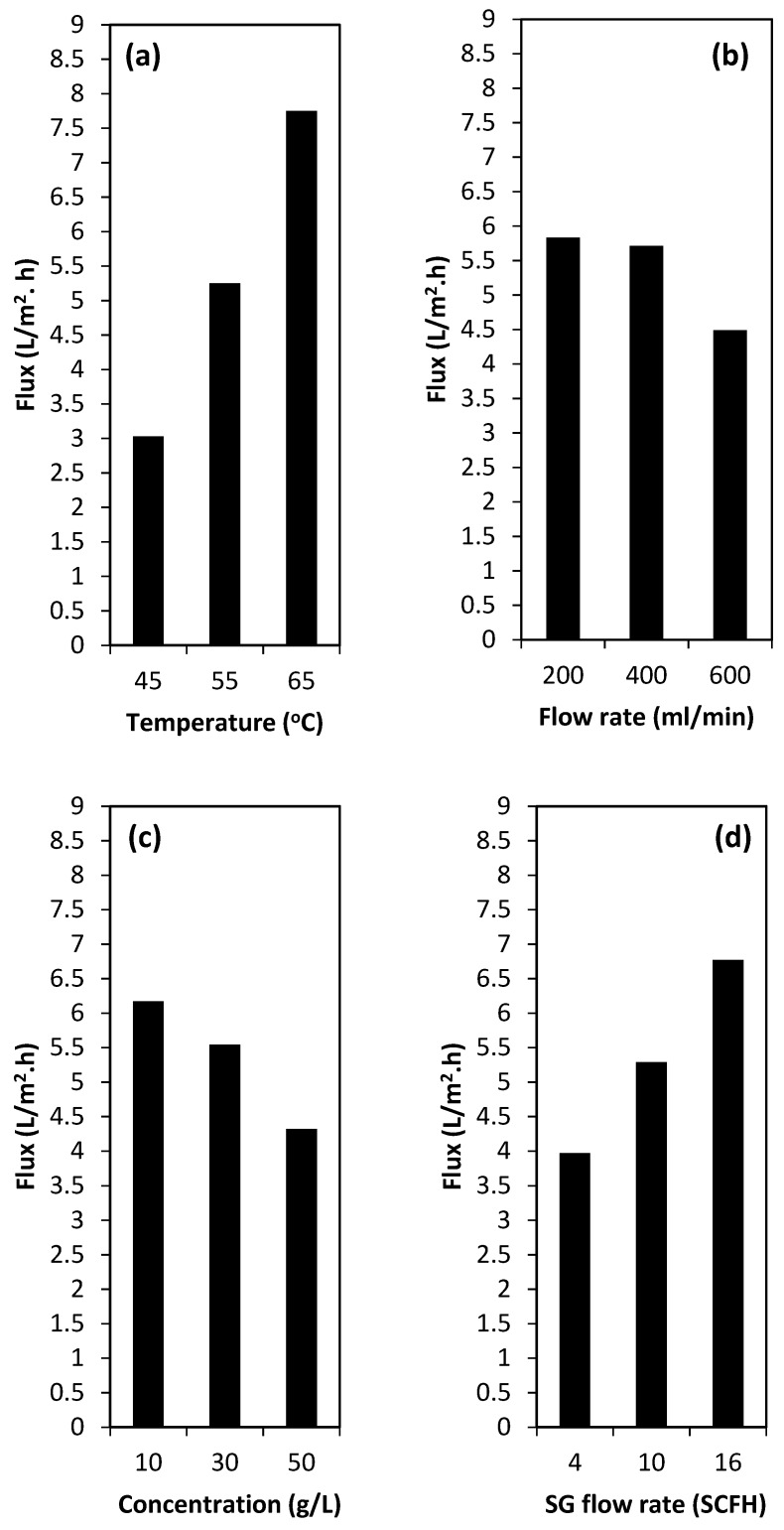
The main effects of feed temperature (**a**), feed flow rate (**b**), feed concentration (**c**), and sweeping gas flow rate (**d**) on the permeate flux based on the operating conditions from the Table 3.

The steady-state condition of the system was achieved using both distilled water and glucose syrup for about 1 h. The permeate fluxes were reported after this time. An *L*_9_ orthogonal array (four variables in three levels) was offered by Taguchi design methodology. Table 3 represents the experimental variables and their levels. Each row represents a specific experiment. Figure 3a–d shows the main effect of the operating variables on the permeate flux.

### 3.1. Main Effects of the Operating Parameters

In this work, the effect of investigated operating parameter of the SGMD process on the permeate flux at different levels (see Table 3) were plotted, separately. This is due to the experimental design which was orthogonal. Figure 3 shows the response value for each level.

Feed temperature (°C) was investigated as the first operating variable. This was due to the nature of the SGMD process which is a non-isothermal separation process. In the SGMD process, the driving force is a function of the temperature difference (Δ*T*) between the two sides of the membrane pores [19]. As can be observed in Figure 3a, increasing the feed temperature increases the permeate flux, considerably. This can be attributed to the higher vapor pressure in the higher feed temperature. Due to the exponential behavior of the temperature versus the vapor pressure, increasing the feed temperature from 55 to 65 °C proved to have a greater effect than raising it from 45 to 55 °C (see Table 4). It is worth noting that when further increasing the vapor pressure in higher operating temperatures, both the temperature and concentration polarizations increased [20]. Moreover, higher feed temperature can lead to higher heat conduction through the membrane; however, this can be highlighted even more in the DCMD process. Hence, higher temperatures cannot necessarily lead to higher permeate fluxes. Moreover, the results presented in Table 4 indicate that there are some interactions among investigated variables. Therefore, the response of each parameter versus the others should be constructed.

Furthermore, the Millard reaction, which is one the major draw-backs in sugar processing [21], is more probable at higher temperatures; hence, the 65 °C was the maximum investigated value for the operating temperature. Moreover, it should be noted that the energy consumption needed to increase the temperature at lower feed temperatures (from 45 to 55 °C in this work) is higher [22,23]; therefore, based on the available energy resource, 65 °C was suggested as the most sufficient operating temperature for concentrating the glucose syrup.

As stated before, the SGMD process is a vapor pressure driven separation. The temperature difference imposes the driving force between the two sides of the membrane, including the feed channel (where the hot feed re-circulates in direct contact with the membrane active layer) and the permeate channel (where vaporization of liquid molecules were carried out when they crossed the membrane pores). Therefore, if the feed flow rate increases too much, the heat transfer between hot feed and cold air will also increase. This means the feed temperature decreases and the temperature of the sweeping air increases [24]. Consequently, this can cause a reduction in the permeate flux [25], and this is also experimentally confirmed in this work (see Figure 3b). As most SGMD experiments carried out using hydrophobic porous membranes were specifically manufactured for microfiltration (MF) applications, designing and developing specific membranes for SGMD applications can solve this draw-back. Although less membrane fouling is expected by using higher feed flow rates, it may decrease the permeate flux, as is shown in Figure 3. This also can be explained by the fact that higher feed flow rates under the constant feed channel depth needs higher inlet pressure. This higher inlet pressure can be translated to the fact of higher pore wetting risk, which consequently can lead to permeate flux decline.

In general, high concentration of solute in the feed stream has an almost negative effect on membrane separations due to the increase in the concentration polarization [26] and vapor pressure reduction (due to the reduction of water activity in aqueous solutions [27]) in MD process. Like other membrane separations, the SGMD process is also sensitive to the concentration polarization, as discussed in the literature [19]. Although this sensitivity is less in the case of the SGMD process; however, the effect of feed concentration on the permeate flux should be studied. As observed in the results of the present work, with increasing the feed concentration, the permeate flux decreases, slightly. This can be attributed to the vapor pressure decline with increasing the glucose concentration in the feed stream. Moreover, higher feed concentration can highlight the effect of the concentration polarization layer (see Figure 3c). It should be noted that one of the most important advantages of the SGMD process in comparison with other membrane separations, which use pressure difference for concentrating sugar syrups, such as the ultrafiltration (UF) and reverse osmosis (RO) process [26], is that the MD process is not sensitive to osmotic pressure limitations. Even though the flux reduction at higher feed concentrations occurs, the MD process can be used for dewatering of the highly concentrated sugar syrups. Figure 3c presents the effect of feed concentration of the target parameter, i.e., the permeate flux.

Figure 3d shows the main effect of the sweeping gas (SG) flow rate on the permeate flux. As can be seen, the SG flow rate can affect the permeate flux, significantly. The higher the sweeping gas flow rates, the higher the fluxes achieved (see Figure 3d). This could be explained as follow. Higher SG flow rate can lead to the vapor pressure reductions in the cold stream (permeate side), which can impose higher mass transfer (larger driving force). Moreover, increasing the SG flow rates can significantly decrease the temperature polarization effect in the cold stream of the MD module. As in the SGMD process, there is no pore wetting risk from the permeate side, the SG flow rate can be as high as possible. However, based on the obtained results it can be recommended that the gas inlet pressure should be lower than that of the hot stream inlet pressure.

### 3.2. Interactions of Variables

**Figure 4:**
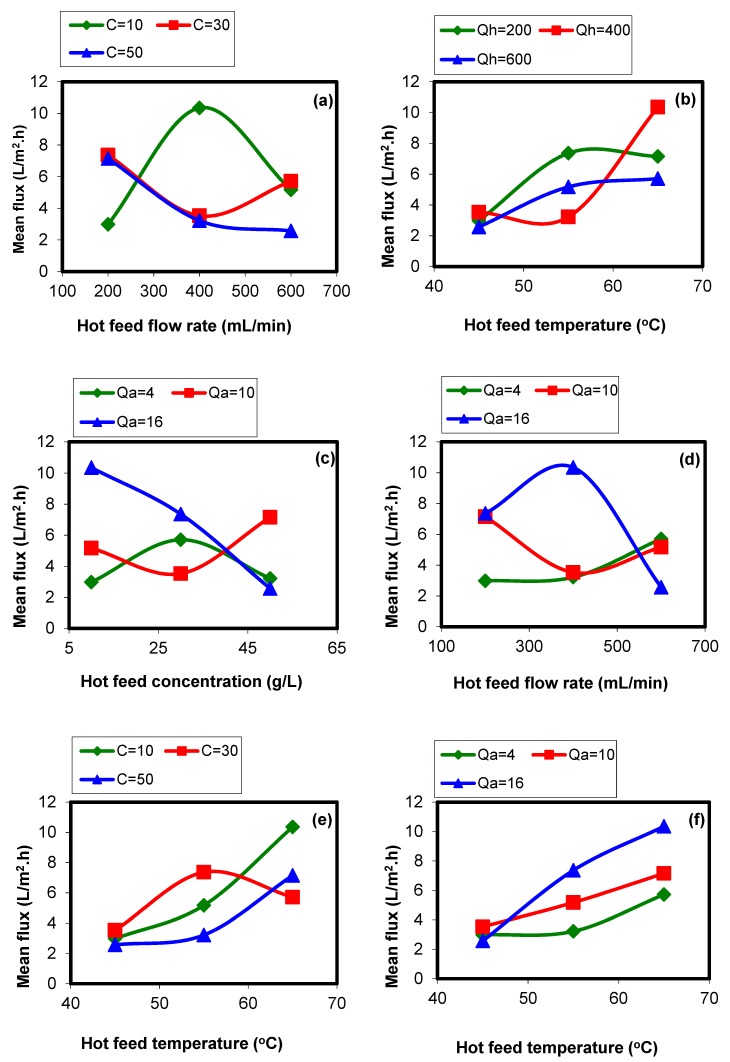

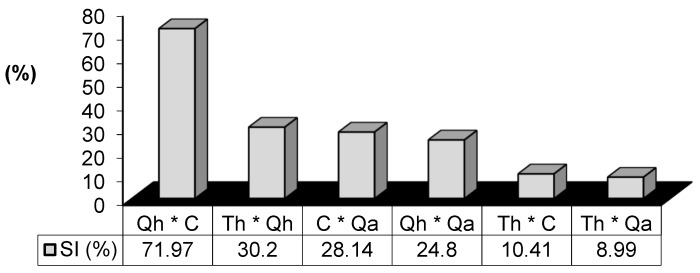
Interactions between the operating variables ((**a**): *Q_h_* versus *C*; (**b**): *T_h_* versus *Q_h_*; (**c**): *C* versus *Q_a_*; (**d**): *Q_h_* versus *Q_a_*; (**e**): *T_h_* versus *C*; and (**f**): *T_h_* versus *Q_a_*) and their related severity index (SI%) based on the operating conditions from Table 3.

The experimental results indicated that there are some interactions between the parameters. To identify the interaction between the parameters, the response of each parameter against the others should be plotted [27]. The results of interactions are shown in Figure 4. As could be observed, the regions in which there are interactions are illustrated. In each graph in which the lines with different colors (three level for each parameter) cross each other there is an interaction between the parameters.

These interactions are the result of the polarization phenomena in the system. Moreover, the module design can significantly affect the interaction among operating variables. In this work, a plate and frame module with 130 × 130 mm^2^ dimensions was used for the SGMD experiments. In some cases, the investigated geometry can make these interactions severer, especially when the DCMD configuration is used because the both sides are in direct contact with the process liquids (temperature polarization is probable in both feed and permeate sides). However, for the SGMD configuration, a lower temperature polarization effect is expected on the permeate side, while the negative effect on the feed side can still exist.

(*T_h_*: feed temperature, *Q_h_*: feed flow rate, *C*: feed concentration, and *Q_a_*: sweeping gas flow rate).

The results of the interaction study (see Figure 4) show that the higher interaction level was observed for *Q_h_* (feed flow rate) and *C* (feed concentration) with the severity index (SI) of 71.97%. This can be attributed to the effects of concentration polarization, temperature polarization, and heat loss through the membrane body (the thermal conduction). It can be concluded that the *Q_h_* and *C* had almost the same effect on the system response (permeate flux). Based on the obtained results, low feed flow rate, and high feed concentration, both can intensify the effect of concentration and temperature polarizations, as well as conductive heat loss through the membrane’s body. The interaction between *T_h_* (feed temperature) and *Q_h_* with the severity index of 30.2% is also the result of the temperature polarization. This is true for the other interactions.

On the other hand, the minimum interaction imposes between the feed temperature and the SG flow rate (SI = 8.99%). The feed temperature imposes its effect in the feed channel while the SG flow rate affects the permeate side and these regions are separated by the applied membrane. However, both of these parameters are effective on the permeate flux, separately.

### 3.3. Analysis of Variance (ANOVA)

Using the analysis of variance (ANOVA), the effect of operating parameters on the permeate flux can be investigated, significantly. This is followed up by separating the total variability of each level. This is measured by the sum of the squared (*S*) deviations from the total mean of the responses, into contribution by each SGMD process parameter and the error [28]. In addition, the importance of SGMD process parameters can be evaluated by the percentage contribution by each of the process parameters in the total sum of the squared deviations.

According to the ANOVA result for the operating parameters of the SGMD process the most significant operating variable(s) affecting the performance characteristic (i.e., the permeate flux as the target parameter in this work) can be investigated. The ANOVA results, which have been shown in Table 5 indicate that the feed temperature and the feed flow rate are the most and the least significant operating variables due to their higher and lower percentages of contribution (62.14% and 6.11%, respectively). As could be observed, the degree of freedom (DOF) for all variables is 2 and *F*-ratio is zero, based on the obtained results and experimental design in this study.

Moreover, based on the pool-factor analysis, all studied operating variables were effective. The error for these experiments was very low which indicates the accuracy of the performed tests and obtained experimental results.

### 3.4. Taguchi Model Validation

**Figure 5:**
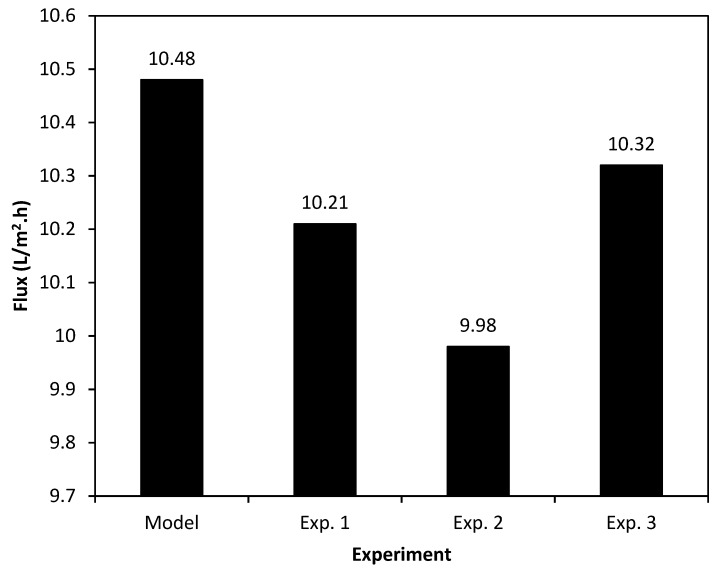
The average permeate flux based on the results of the Taguchi model and experiments after 8 h under the optimum operating conditions (*T_h_*: 65 °C, *Q_h_*: 200 mL/min, *C*: 10 g/L, and *Q_a_*: 16 SCFH).

The optimum operating conditions can be predicted using the Taguchi method after analyzing the proposed experimental results. Moreover, it reports the expected target parameter, i.e., permeate flux, at the proposed optimum operating conditions. Table 6 shows the results of this work. According to Taguchi method, the best combination of the SGMD process parameters is *A*_3_*B*_1_*C*_1_*D*_3_. Using this combination of the operating parameters, the highest permeate flux will be attainable. The *A*_3_*B*_1_*C*_1_*D*_3_ refers to feed temperature of 65 °C, feed flow rate of 200 ml/min, sugar concentration of 10 g/l, and SG flow rate of 16 SCFH, respectively. The predicted permeate flux under the proposed conditions was measured at 10.48 L/m^2^·h. In order to assure the validity of the prediction of the Taguchi model, three individual experiments were carried out using the *A*_3_*B*_1_*C*_1_*D*_3_ experimental conditions in different times. Figure 5 shows the results of the Taguchi analysis prediction and the conducted experiments. Although a good agreement between the Taguchi prediction and the conducted experimental results can be observed (see Figure 5), the obtained experimental permeate fluxes were slightly lower than that of the Taguchi prediction. Under the real operating conditions and at different test times there were some noises from the environment, such as humidity and temperature variation which can affect the permeate flux. These noses were investigated by the Taguchi method. That is why the predicted permeate flux was slightly higher than that of the test results.

Furthermore, Table 7 shows the final concentrations and separation percentages for each experiment after 3 h of operations.

### 3.5. SGMD Performance Evaluation

The results of experiments including the separation factor (%*S*) and the obtained flux (L·m^−2^·h^−1^) for each test are summarized in Table 3. As can be observed, the lowest and the highest separation factors were achieved for the lowest glucose concentration in the feed (10 g/L), i.e., 25.25% (test 1) and 82.95% (test 8), respectively. The results confirm that the feed temperature was the most effective operating parameter. This is in good agreement with the literature [29,30,31,32]. The highest investigated operating temperature in this study was 65 °C. This temperature was not only in the safe range for processing the sugar syrup (due to thermal sensitivity of the glucose), but was also achieved by using the waste heat in the industrial sectors or by solar heating. The ability of coupling waste heat or solar energy is one of the most important advantages of all MD configurations. Moreover, the 99% solute rejection was achieved for experimental tests in this study.

Table 7 compares the results of this work and the results of the literature. It should be noted that the some of these studies have used the sucrose syrup as the feed sample. Among the MD configurations, the DCMD is the most investigated one for different applications, specifically desalination [32]. However, due to the presence of process liquids in both the feed and permeate channels, the pore wetting risk is considerable [33]. The same concern is attributed to the VMD process, while this configuration is more practical for removing volatile components. On the other hand, the obtained permeate flux using the AGMD process was not high enough for the concentration of the sugar syrup (see Table 7). However, in the SGMD process, only one side of the applied membrane was in direct contact with the process liquid, i.e., in the feed channel. This can considerably lower the pore wetting risk, and consequently make the SGMD process a promising alternative for separations in which the permeance is not the target product. Comparing the results of published data from the literature for concentrating different sugar syrups can also confirm this conclusion. As can be observed in Table 7, the permeate flux of the SGMD configuration was more affordable compared with the other MD configurations.

## 4. Conclusions

In this work, the Taguchi experimental design and optimization were investigated for the sensitivity analysis of concentrating the glucose syrups using the SGMD process. Feed temperature and sweeping gas flow rate showed the main effects on the permeate flux. Based on the available energy source, feed temperature at the range of 55 °C to 65 °C is suggested for sugar processing. Results indicated that increasing the feed concentration can lead to a reduction in the permeate flux. Based on the pool-factor analysis, all selected variables were effective. The Taguchi method predicted that the best operating conditions could be achieved using the third, the first, and again the third levels for temperature, feed flow rate and concentration, and sweeping gas flow rate, respectively. Overall, it can be concluded that the SGMD process with a high level of solute rejection (99% in this work for all conducted tests) can be effectively used for the sugar syrup concentration step in bioethanol processing.

## Figures and Tables

**Table 1:** Introduction and explanation of different configurations of membrane distillation (MD) process [14,15,16,17].

Configuration	General Scheme	Specification	Description
DCMD	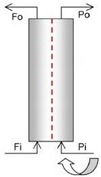	• Both sides of the membrane are in direct contact with the hot and cooling streams	High-permeate flux Low-energy efficiency The simplest operation The most used configuration Highest conduction lost
AGMD	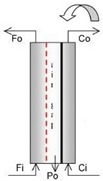	• A stagnant air-gap in the distillate side is interposed between the membrane and a highly heat-conductive condensing plate	Highest energy efficiency Low distillate flux The air-gap distance can be 2–10 mm
SGMD	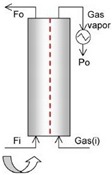	• Cold inert gas or air stream is used as carrier for stripping the molecules	Mostly practical for concentrating purposes Condensation happens outside the module
VMD	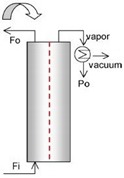	• Permeate channel is under vacuum	Mostly practical for volatiles removal Vapor molecules are condensed outside the module

**Table 2:** Characteristics of the membrane applied in this study.

Type	PTFE
Pore size (micron)	0.22
Porosity (%)	70
Thickness (micron)	175
Contact angle (°)	132.5 ^†^
Average roughness (nm)	71 ^†^

^†^ Measured value in this work. PTFE: Polytetrafluoroethylene

**Table 3:** Operating variables and their levels based on the Taguchi *L*_9_ orthogonal array, as well as the corresponding permeate flux for each test.

Test No.	*T_h_* (°C)	*Q_h_* (ml/min)	*C_i_* (g/l)	*Q_a_* (SCFH)	*S* (%)	*Flux* (L·m^−2^·h^−1^)
1	45	200	10	4	25.25	2.98
2	45	400	30	10	31.03	3.54
3	45	600	50	16	24.50	2.58
4	55	200	30	16	55.04	7.36
5	55	400	50	4	29.15	3.22
6	55	600	10	10	41.40	5.19
7	65	200	50	10	68.50	7.16
8	65	400	10	16	82.95	10.36
9	65	600	30	4	42.59	5.72

**Table 4:** Responses for the Taguchi analysis of the permeate fluxes; *T_h_* (feed temperature), *Q_h_* (feed flow rate), *C* (feed concentration), and *Q_a_* (sweeping gas flow rate).

Responses	Process Variables			
	*T_h_*	*Q_h_*	*C_i_*	*Q_a_*
L_1_	3.033	5.833	6.173	3.937
L_2_	5.233	5.706	5.539	5.293
L_3_	7.746	4.493	4.320	6.766
L_2_–L_1_	2.22	− 0.127	− 0.635	1.32
L_1_–L_2_	−2.221	0.126	0.634	−1.321
L_1_–L_3_	−4.714	1.339	1.825	−2.794
L_2–_L_3_	−2.494	1.213	1.218	−1.473
L_3_–L_1_	4.713	−1.340	−1.853	2.793
L_3_–L_2_	2.493	−1.214	−1.219	1.472

*T_h_*: feed temperature; *Q_h_*: feed flow rate; *C*: feed concentration; *Q_a_*: sweeping gas flow rate.

**Table 5:** The results of analysis of variance (ANOVA).

Factor	DOF (*f*)	Sum of Squares	Variance	Contribution Percent *P* (%)
*T_h_*	2	33.36	16.68	62.14
*Q_h_*	2	3.28	1.641	6.11
*C*	2	5.32	2.66	9.91
*Q_a_*	2	11.71	5.85	21.82
Other/Error	0	-	-	0.003
Total	8	53.68	-	99.99%

*T_h_*: Feed temperature; *Q_h_*: Feed flow rate; *C*: Feed concentration; and *Q_a_*: Sweeping gas flow rate.

**Table 6:** Optimized operating conditions and predicted distillate flux based on the Taguchi method.

Factor	Level	Value
*T_h_* (A)	3	65 °C
*Q_h_* (B)	1	200 mL/min
*C* (C)	1	10 g/L
*Q_a_* (D)	3	16 SCFH
Expected result for permeate flux at optimum conditions		10.484 (L/m^2^·h)

**Table 7:** Experimental results of this work and literature data for concentrating of sugar syrup using the MD processes.

Year	Feed	Configuration	Conditions	Flux (L·m^−2^·h^−1^)	Ref.
1999	Sucrose syrup	AGMD	*T_h_*: 42.5 °C Feed flow rate: 43.3 L/h *C_i_*: 90–300 g/L Membrane: PVDF (0.2 and 0.45 µm) and PTFE (0.2 and 0.45 µm) Module: Plate and frame	8.3	[29]
2012	Lignocellulosic hydrolyzates (Glucose)	VMD	*T_h_*: 65 °C *Q_h_*: 1 m/s *P*: 5 kPa Membrane: PVDF (0.18 µm) Module: Hollow-fiber	8.46	[30]
2019	Glucose syrup	SGMD	*T_h_*: 65 °C *Q_h_*: 400 mL/min SG flow rate: 16 SCFH Membrane: PTFE (0.22 µm) Module: Plate and frame	10.36	Present work

## Nomenclature

MD	Membrane distillation
SGMD	Sweeping gas membrane distillation
*T_h_*	Feed temperature (°C)
*Q_h_*	Feed flowrate (mL/min)
*C_i_*	Feed concentration (g/L)
*Q_a_*	Sweeping gas flowrate (SCFH)
PTFE	Polytetrafluoroethylene
*F_D_*	Permeate flux (L·m^−2^·h^−1^)
*S*	Selectivity (%)
SG	Sweeping gas
SI	Severity index (%)
